# Supplementary material for: From sequence to enzyme mechanism using multi-label machine learning
Source: BMC Bioinformatics. 2014 May 19;15:150. doi: 10.1186/1471-2105-15-150 (PMC4229970; doi:10.1186/1471-2105-15-150)
Supplement: Additional file 2 — Java code of ml2db. Additional file ml2db_code.tar.gz contains the Java source code to run the multi-label machine learning experiments and save the results to database. The code’s Javadoc is included. [file 1471-2105-15-150-S2.zip › additional file 2/ml2db/utils/doc/uk/ac/standrews/utils/main/stats/package-tree.html]

uk.ac.standrews.utils.main.stats Class Hierarchy


JavaScript is disabled on your browser.


- Overview
- Package
- Class
- Use
- Tree
- Deprecated
- Index
- Help

- Prev
- Next

- Frames
- No Frames

- All Classes

# Hierarchy For Package uk.ac.standrews.utils.main.stats

Package Hierarchies:

- All Packages

## Class Hierarchy

- java.lang.Object
  - cern.colt.PersistentObject (implements java.lang.Cloneable, java.io.Serializable)
    - cern.jet.random.AbstractDistribution (implements cern.colt.function.DoubleFunction, cern.colt.function.IntFunction)
      - cern.jet.random.AbstractContinousDistribution
        - cern.jet.random.Pareto
          - uk.ac.standrews.utils.main.stats.TruncatedPareto
            - uk.ac.standrews.utils.main.stats.PseudoTruncatedPareto
  - uk.ac.standrews.utils.main.stats.PseudoTruncatedParetoSingleton
  - uk.ac.standrews.utils.main.stats.StatUtils
  - uk.ac.standrews.utils.main.stats.UniformRandomSingleton
  - uk.ac.standrews.utils.main.stats.UniformRandomUtils

- Overview
- Package
- Class
- Use
- Tree
- Deprecated
- Index
- Help

- Prev
- Next

- Frames
- No Frames

- All Classes
